# Biomarkers of Hypoxic-Ischemic Encephalopathy in Newborns

**DOI:** 10.3389/fneur.2012.00144

**Published:** 2012-11-02

**Authors:** Martha Douglas-Escobar, Michael D. Weiss

**Affiliations:** ^1^Department of Pediatrics, University of FloridaGainesville, FL, USA; ^2^McKnight Brain Institute, University of FloridaGainesville, FL, USA

**Keywords:** biomarkers, hypoxic-ischemic encephalopathy, brain injury

## Abstract

As neonatal intensive care has evolved, the focus has shifted from improving mortality alone to an effort to improve both mortality and morbidity. The most frequent source of neonatal brain injury occurs as a result of hypoxic-ischemic injury. Hypoxic-ischemic injury occurs in about 2 of 1,000 full-term infants and severe injured infants will have lifetime disabilities and neurodevelopmental delays. Most recently, remarkable efforts toward neuroprotection have been started with the advent of therapeutic hypothermia and a key step in the evolution of neonatal neuroprotection is the discovery of biomarkers that enable the clinician-scientist to screen infants for brain injury, monitor progression of disease, identify injured brain regions, and assess efficacy of neuroprotective clinical trials. Lastly, biomarkers offer great hope identifying when an injury occurred shedding light on the potential pathophysiology and the most effective therapy. In this article, we will review biomarkers of HIE including S100B, neuron specific enolase, umbilical cord IL-6, CK-BB, GFAP, myelin basic protein, UCHL-1, and pNF-H. We hope to contribute to the awareness, validation, and clinical use of established as well as novel neonatal brain injury biomarkers.

## Introduction

Biomarkers are molecules released by or specific to a particular organ, can give a glimpse into the physiologic or pathologic status of that specific organ (Ling and Sylvester, 2011). Biomarkers can be obtained from the blood, urine, cerebrospinal fluid (CSF), or any other bodily fluid. In neonates with brain injury, biomarkers may be able to predict the degree and location of injury shortly after the injury occurs. The discovery of neonatal brain injury biomarkers is a key step in neonatal neuroprotection. Biomarkers may enable the clinician-scientist to screen infants for brain injury, monitor the progression of disease, identify injured brain regions, and assess the efficacy of neuroprotective strategies procedures in clinical trials. In addition, large-scale validation of the potential biomarkers is required, because the potential confounders (especially for biomarkers that are non-organ specific such as inflammatory mediators). Currently, clinicians do not routinely use biomarkers to care for neonates with brain injuries. This review will examine potential biomarkers the bedside clinician-scientist may use to hone the treatment of neonates with hypoxic-ischemic encephalopathy.

## Hypoxic-Ischemic Encephalopathy

Systemic asphyxia manifests in the brain as hypoxic-ischemic encephalopathy (HIE; Vannucci, 1997). Systemic asphyxia occurs in about 2% of full-term infants and in nearly 60% of very low birth weight (premature) newborns (Mulligan et al., 1980; Giffard et al., 1990; Low et al., 1997). Twenty to fifty percent of asphyxiated babies who exhibit severe HIE die during the newborn period (MacDonald et al., 1980). Of the survivors of severe HIE, up to 25% have permanent neuropsychological handicaps in the form of learning disabilities, epilepsy, cerebral palsy, with or without associated mental retardation, learning disabilities, or epilepsy (Finer et al., 1981; Robertson et al., 1989). Systemic asphyxia that causes HIE may occur prior to delivery (e.g., placental abruption, toxemia, maternal collagen vascular disease), during delivery (e.g., prolonged labor, difficult delivery, abnormal presentation), or after delivery (e.g., sepsis, shock, respiratory distress). Currently, hypoxic-ischemic injury is diagnosed based on clinical criteria. This review will use the term HIE although recently medical experts have proposed use the term neonatal encephalopathy instead of HIE.

A clinician’s ability to predict the outcome of neonates with HIE is not straightforward. The Sarnat grading system (Sarnat and Sarnat, 1976) stages HIE based on clinical criteria. This scoring system divides neonates into mild, moderate, or severe categories, and measures the progression of the neurologic insult to predict a neonate’s prognosis (Finer et al., 1981). Nevertheless, the Sarnat score system is subjective and changes over time. A new bedside tool, amplitude integrated electroencephalogram (aEEG), may help stage the severity of injury and predict prognosis (Hellstrom-Westas et al., 1995). Unfortunately, the Sarnat score and aEEG are not as effective in predicting outcomes in neonates during hypothermia (Thoresen et al., 2010) and do not provide information about the timing of the injury. Brain MRI can help determine when the injury occurred, but obtaining an MRI is not possible in unstable patients.

Recently, therapeutic hypothermia has evolved into standard of care for neonates with moderate to severe HIE. Prior to this therapy, neonates were treated with systemic supportive care with no specific therapy aimed at preventing or ameliorating ongoing brain injury. Large randomized multicenter trials demonstrated that hypothermia in neonates with moderate to severe HIE is safe, improves outcomes, and has a combined number needed to treat of one in nine (Gluckman et al., 2005; Azzopardi et al., 2009). The next step in brain neuroprotection is the identification of biomarkers that can facilitate clinical decisions. Biomarkers will help clinicians identify neonates that will respond to hypothermia and those that will need other new neuroprotective interventions. If clinicians are able to stratify patients using biomarkers, neonates will be protected from exposure to unnecessary, ineffective therapies. Furthermore, these same infants may benefit from other specific therapies more tailored to their biological profile. Biomarkers will be a key feature of future neuroprotective trials and will help gage the intervention’s short- and long-term efficacy.

### Biomarkers of hypoxic-ischemic encephalopathy

To date, potential biomarkers have been identified in neonates with HIE. These biomarkers were obtained from CSF, serum, and urine and include S100B, neuron specific enolase (NSE), umbilical cord Interleukin-6 (IL-6), CPK-BB, glial fibrillary acidic protein (GFAP), myelin basic protein, Ubiquitin carboxyl-terminal hydrolase L1 (UCHL-1), and pNF-H (see Table 1).

**Table 1:** **Summary of biomarkers characteristics**.

Biomarker	Description	Cell specificity	Pathophysiology of high plasma concentrations
*BDNF*	Neurotrophic factor	Secreted by *neural progenitor stem cells, astrocytes, and neurons*. There are trace amounts in platelets	Released after brain injury (neuronal and astrocyte cell death) but concentration can be altered by exercise, depression, and autoimmune disease
*S100*β	It is a protein that binds calcium and is a major component of the cytosol in various cell types	*Astroglial* cells have a high concentration of S100B. Other cells can release S100B	Released predominantly after astrocyte death but can be released from other tissue damage
*GFAP*	It is a cytoskeletal intermediate filament protein found in the astrocytes	Specific marker of *differentiated astrocytes*	Released after astrocyte death
*NSE* or neuron specific enolase or enolase 2	Glycolytic isoenzyme (γγ)	High concentrations of NSE are found in mature central and peripheral *neurons*. Although there are trace amounts of similar isoenzyme (αγ) in platelets	Released after neuronal death

*Summarizes main biomarkers for hypoxic-ischemic encephalopathy including its description, cell specificity, and pathophysiology of high plasma concentrations*.

As discussed above, a primary goal of biomarkers is to identify injury and predict long-term outcomes. The best sources for biomarkers in critically ill neonates are those fluids obtained the least invasively. Therefore, an ideal biomarker would come from the urine or saliva. Ideally, biomarkers could be collected shortly after birth and help to determine the time at which the hypoxic-ischemic injury occurred and predict the neonate’s outcome. Counter-intuitively, biomarkers that do not originate from brain could be good predictors of outcomes such as death and long-term neurodevelopmental handicaps. For example, IL-6 is an inflammatory cytokine produced by T-cells and macrophages, and was found by Chiesa et al. (2003) to be 376-fold higher in 50 infants without infection who developed HIE compared to 113 normal infants. The IL-6 concentrations was 5.5-fold higher in the HIE infants than the asphyxiated newborns without HIE. In addition IL-6 concentrations were significantly related to the severity of HIE and the neurodevelopmental outcome at 2 years of age. Maternal serum IL-6 concentration did not correlate with the risk of neonatal HIE.

**S-100** is a calcium binding protein and is a major component of the cytosol in various cell types. In particular, glial cells have a high concentration of S100B. S100B immunoassay kits are commercially available and can detect S100B in many biological fluids (urine, blood, CSF, amniotic fluid, saliva, and milk; Gazzolo and Michetti, 2010; Gazzolo et al., 2010). Furthermore, reference ranges are available for newborns and children through age three (Bouvier et al., 2011) and urine S100B reference ranges for preterm and term healthy newborns (Gazzolo et al., 2007). Serum S100B concentrations in healthy children are higher than concentrations reported in adults. These serum concentrations decrease over time, especially during the first 6 months after birth. Similarly, urinary S100B protein concentrations are higher in premature infants than in term newborns and steadily decrease with advancing GA.

Gazzolo et al. (2004) demonstrated that S100B concentrations in the first urine after birth were significantly higher in HIE patients than in controls. S100B has been investigated in cord blood samples and has been linked to HIE. Cord blood of 40 neonates with HIE had elevated S100B protein concentrations when compared with controls (Qian et al., 2009). In the same study, concentrations of S100B greater than 2.02 μg/L had a sensitivity of 86.7% and a specificity of 88% for predicting the development of moderate or severe HIE.

Gazzolo et al. (2009) also demonstrated that an S100B concentration cut-off of 0.41 mcg/L had a sensitivity of 91.3% and a specificity of 94.6% for predicting the development of HIE. The sensitivity and specificity increased to 100 and 98.8%, respectively, when urine samples were collected at 4–72 h after birth. In another study of 132 term infants, urinary S100B concentrations were higher in infants who suffered perinatal asphyxia or died and urine S100B above 1 mcg/L predicted neonatal death with a sensitivity and specificity of 100%. A study by the same group demonstrated that urinary S100B concentrations were not affected by renal failure (Risso et al., 2011).

Glial fibrillary acidic protein is a cytoskeleton intermediate filament protein of the astrocytes and is only released into the blood upon astrocyte death. GFAP have been correlated with poor outcomes in adult patients after stroke, cardiac arrest, or traumatic brain injury (Pelinka et al., 2004a). GFAP has been used as a predictor of mortality or poor neurological outcomes in children requiring extracorporeal membrane oxygenation (Pelinka et al., 2004b; Vos et al., 2004; Lumpkins et al., 2008; Kaneko et al., 2009; Bembea et al., 2010). A recently published pilot study compared 23 HIE neonates who met the criteria for hypothermia with 23 NICU patients without neurologic injury (Ennen et al., 2011). The patients with HIE had significantly elevated GFAP concentrations when compared with controls. In addition, a GFAP equal to or greater than 0.15 ng/mL upon NICU admission was predictive of an abnormal brain MRI.

Other serum biomarkers have been explored to predict long-term neurologic deficits after neonatal asphyxia. In a recent meta-analysis, Ramaswamy et al. (2009) pooled data from published studies of neonatal HIE biomarkers that followed patients beyond 12 months of age. Serum and CSF concentrations of IL-1b, IL-6, and serum NSE were predictive of abnormal outcomes. In addition, high GFAP concentrations in CSF were predictive of death.

Neuron specific enolase belongs to the family of enolases, enzymes present in all tissues and organisms capable of glycolysis. Enolases have three subunits (α, β, and γ) each one encoded by separate genes. The subunits can combine to form five different isoenzymes: αα, αβ, αγ, ββ, and γγ. Enolase 1 (αα) is found in liver, kidney, spleen, and adipose tissue. Enolase 3 (ββ) is muscle specific enolase. Enolase 2 (γγ) is NSE found in central and peripheral neurons and neuroendocrine cells. The mature neurons and glia can be distinguished by the content of enolase: neurons only have NSE and glia express enolase 1 (Marangos et al., 1980a). Minimal quantities of enolase can be found in platelets (0.045% of the total soluble protein of platelets); nevertheless most of the enolase found in platelets is αγ subunits (Marangos et al., 1980b). High levels of NSE in CSF and serum are correlated with poor outcome in patients with cardiac arrest (Roine et al., 1989; Rundgren et al., 2009), in patients with cerebrovascular accident (Hay et al., 1984) and pediatric patients with traumatic brain injury (Berger et al., 2005). Detection of NSE in peripheral serum is only expected to occur after both, neuronal death and disruption of the blood brain barrier. Animal models (Costine et al., 2012) have demonstrated a correlation between the volume of cortical injury and levels of NSE following a traumatic brain injury. Elevated serum NSE concentrations in neonates undergoing cardiac surgery correlate with poor prognosis even when parallel samples of CSF do not reveal elevated NSE levels (Schmitt et al., 1998).

Celtik et al. (2004) explored serum neuron specific enolase as a predictor of HIE severity. According to ROC curves, serum NSE above 40 mcg/L obtained between 4 and 48 h could distinguish infants with no or mild HIE from infants with moderate or severe HIE. Additionally, serum NSE concentrations with a cut-off point of 45.4 mcg/L could distinguish infants with poor outcomes from infants with normal outcomes.

Analyses of brain MRIs in patients with HIE have identified the most common patterns of brain injury: basal ganglia injury, diffuse or focal cortical injury, and injury to watershed areas of the cortex. Two studies have attempted to correlate biomarkers of HIE with various MRI patterns of brain injury. Ennen et al. (2011) found that high serum GFAP concentrations in the first 2 days of life in neonates undergoing whole body hypothermia correlate with abnormal brain. Douglas-Escobar et al. (2010) measured serum **UCHL-1** (found in neuronal cell bodies) and **pNF-H1** (found in white matter brain regions) in patients with severe HIE and controls. Correlations were found between the serum levels and the MRI patterns of injury. Both studies were pilot studies with very low patient numbers therefore need further validation. The ability to predict the outcomes of HIE patients may be improved when biomarkers are used in combination with brain MRI. For example, combining trajectory of biomarkers such as NSE with MRI, improved the long-term prognostic prediction (Berger et al., 2010).

A final interesting category of potential biomarkers is the neurotrophins. Brain derived neurotrophic factor (BDNF) is a neurotrophin that binds to the TrkB and p75^NTR^ receptors. BDNF supports the survival of existing neurons and encourages the growth and differentiation of new neurons and synapses. Imam et al. (2009) described higher cord plasma BDNF levels in newborns with HIE when compared with control neonates. These elevated BDNF levels predicted poor neurologic outcomes. Our laboratory has found evidence that brain BDNF concentrations are increased after rodent model of neonatal HI, similar to reports by others in the post-stroke milieu (Bejot et al., 2011). Researchers have postulated that BDNF increases the migration of stem cells (Borghesani et al., 2002). We can speculate from animal models, the high plasma concentrations of BDNF are reflection of high brain BDNF concentrations released by neural progenitor cells and astroglia cell in an attempt to foster brain cell recovery.

## Future Directions

Hypothermia is the most promising of the neuroprotective therapies that have emerged over the past decade and is rapidly becoming the baseline therapy upon which future neuroprotective agents will be added. However, only one in eight neonates treated with hypothermia respond to the treatment. Biomarkers may help the bedside clinician identify neonates that will responders and non-responders to hypothermia. Non-responder patients could to be selected to add new neuroprotective strategies. Biomarkers may help to determine the time that the injury occurred. This is important, because hypoxic-ischemic injury often begins *in utero and if* too much time has elapsed from the brain injury, neonates would not benefit from treatment with hypothermia. This may explain why some neonates with HIE do not respond to hypothermia. The timing of injury also has major medico-legal ramifications for the obstetric and neonatal team taking care of the infant.

Using a panel of biomarkers for neonatal brain injury also holds the promise of allowing for more individualized care of neonates (see Table 2). For example, certain neonates may have more of an inflammatory component than others. Once identified, these patients could be treated with agents that minimize the inflammatory cascade. Serum levels of biomarkers could also be utilized to monitor the neonate’s response to pharmacologic agents. A decrease in plasma biomarker concentrations could potentially indicate a preservation of endogenous tissue.

**Table 2:** **Biomarkers and their potential use in neonatal brain injury**.

Biomarker	Category	Fluids locations	Associations	Usefulness
S100β	Brain-specific protein	Cord blood, urine, saliva, milk blood, CSF	Pregnancy complicated with growth restriction and trisomy 21	++
			Neonates with asphyxia and HIE	
			Mortality in term newborns	
Interleukin 6	Inflammatory maker	Cord blood and blood	Neonates with HIE	+
GFAP	Brain-specific protein	CSF	Neonates with HIE	+
Neuron specific enolase	Brain-specific protein	Blood and CSF	Neonates with HIE, mortality	+++

*Summarizes potential biomarkers of asphyxia and hypoxic-ischemic encephalopathy (HIE). These biomarkers have been detected in blood, urine, saliva, milk, cerebrospinal fluid (CSF), and brain tissue. Usefulness of the biomarkers: (+) limited use because CSF samples are required, (++) very useful but can be altered by other factors such as gestational age and intrauterine growth restriction, (+++) very useful because it is more specific to brain injury and detected in serum*.

Biomarkers may also be able to identify specific brain regions that undergo injury following HIE. These regions may respond better to a specific treatment. Therefore, in the future panel of biomarkers may be utilized to identify injury to particular brain regions. To date, none of the examined biomarker trials have predicted the aforementioned due to small patient numbers.

*In summary, more studies are needed to correlate and validate the clinical use of possible biomarkers of hypoxic-ischemic brain injury. In the future, more sensitive and precise instruments for brain imaging (such as brain MRI), brain functioning (such as NIRS, aEEG), and long-term neuro-assessment should be incorporated to validation of biomarkers of neonatal brain injury*.

## Conflict of Interest Statement

The authors declare that the research was conducted in the absence of any commercial or financial relationships that could be construed as a potential conflict of interest.

## Abbreviations

aEEG, amplitude integrated electroencephalogram; BDNF, brain derived neurotrophic factor; CPK-BB, brain type creatine phosphokinase; CSF, cerebrospinal fluid; GFAP, glial fibrillary acidic protein; HIE, hypoxic-ischemic encephalopathy; IL-6, interleukin-6; NSE, neuron specific enolase; UCHL-1, ubiquitin carboxyl-terminal hydrolase L1.
